# Priority setting for nutrition research in individuals with spinal cord injury: A protocol for Delphi study among health professionals

**DOI:** 10.1371/journal.pone.0327612

**Published:** 2025-07-11

**Authors:** Marija Glisic, Shashivadan P. Hirani, Hanne Bjørg Slettahjell, Willemijn Faber, Yannis Dionyssiotis, Alastair Forbes, Anthony Twist, Emil Moga, Jackie McRae, Firas Sarhan, Sharon Leigh, Samford Wong

**Affiliations:** 1 Swiss Paraplegic Research, Nottwil, Switzerland; 2 Institute of Social and Preventive Medicine (ISPM), University of Bern, Bern, Switzerland; 3 Department of Global, Public and Population Health and Policy, School of Health and Medical Sciences, City St George’s, University of London, London, United Kingdom; 4 Sunnaas Rehabilitation Hospital, Bjørnemyr, Norway; 5 Department of Nutrition, Institute of Basic Medical Sciences, University of Oslo, Norway; 6 Department of Rehabilitation, Rehabilitation Centre Heliomare, Wijk aan Zee, North Holland, The Netherlands; 7 2nd Physical Medicine and Rehabilitation Department, National Rehabilitation Center EKA, Athens, Greece; 8 Institute of Clinical Medicine, University of Tartu, Tartu, Estonia; 9 Midland Centre for Spinal Injuries, The Robert Jones and Agnes Hunt Orthopaedic Hospital, Gobowen, Oswestry, United Kingdom; 10 National Spinal Injuries Centre, Stoke Mandeville Hospital, Aylesbury, United Kingdom; 11 Department of Allied Health, School of Health and Medical Sciences City St George’s, University of London, London, United Kingdom; 12 Royal Buckinghamshire Hospital, Aylesbury, United Kingdom; Swiss Paraplegic Research, SWITZERLAND

## Abstract

**Study design:**

A protocol for Delphi Consensus Study.

**Objectives:**

To identify a top ten list of priorities for future nutrition research in individuals with spinal cord injury (SCI).

**Setting:**

The International Spinal Cord Society (ISCoS) Nutrition Specialist Interest Group (SIG) priority setting partnership was established in 2024 to conduct this international Delphi study through online surveys and a hybrid meeting.

**Methods:**

The study involves THREE key stages: topic generation, priority ranking, and consensus building. In phase 1, participants will generate potential research topics via an online survey. Phase 2 involves ranking the top 10 research priorities on a 9-point Likert scale. Phase 3 consists of a consensus meeting where stakeholders will engage in discussions and vote on the final priorities using interactive tools. For Phases 1 and 2, both ISCoS Nutrition SIG members and their professional contacts will be invited to participate, ensuring a diverse pool of expertise. Phase 3 will be limited to Nutrition SIG members to facilitate focused decision-making. Data will be collected through secure Qualtrics surveys and analysed using descriptive statistics in STATA or SPSS. The study adheres to the Conducting and Reporting of DElphi Studies (CREDES) recommendations and employs rigorous data management practices compliant with City St George’s, University of London standards.

**Ethics and dissemination:**

Ethics approval has been granted (ref: ETH2425−0192, Health Services Research & Management Proportionate Review Committee, City St George’s, University of London). The findings will be disseminated through ISCoS website, professional conferences and a peer-reviewed journal.

## Introduction

Nutrition plays a pivotal and often underappreciated role in the lives of individuals with spinal cord injury (SCI) profoundly impacting their health, well-being, and overall quality of life [1]. Following an SCI, individuals face a dramatically increased risk of malnutrition, with alarming statistics showing that up to 60% of those admitted to rehabilitation centres experience this potentially debilitating condition [2,3]. This high prevalence is not merely a coincidence but a direct consequence of the complex physiological changes and challenges that accompany SCI. The risk of malnutrition is further exacerbated by a constellation of comorbidities commonly associated with SCI. Dysphagia can impact on nutritional management particularly in acute cervical SCI with the need for temporary or more longer-term enteral nutrition [4]. Neurologic bowel dysfunction, recurrent infections, the presence of pressure injuries, and loss of appetite collectively favour the development of malnutrition [5,6]. The prevalence of malnutrition was reported in the range of 40–69% in individuals with SCI and the consequences of malnutrition in SCI individuals are far-reaching and severe.^3^ Research has consistently demonstrated strong links between malnutrition and poorer functional recovery, significantly prolonged hospitalization periods, and alarmingly higher mortality rates within the first-year post-injury [2,7,8]. These findings underscore the critical need for early nutritional intervention and ongoing management as integral part of SCI care.

Due to lack of standardised recommendation, nutrition service is usually under-invested in SCI centres [9,10]. Unsurprisingly, poor adherence to dietary guidelines is common both in the inpatient and outpatient settings, with two-thirds of individuals consuming insufficient fruits and vegetables while overindulging in meat, and a concerning 10% reporting daily alcohol consumption [11]. This dietary imbalance is not limited to the acute phase; both subacute and chronic SCI populations struggle to meet nutritional recommendations [12–16]. In general, individuals with SCI exhibit a greater energy intake relative to their energy needs, along with an imbalance in fibre intake and macronutrient consumption, characterized by excessive protein and carbohydrate intake. Additionally, there are significant micronutrient deficiencies such as vitamin A, B5, B7, B9, D, E and minerals like potassium and calcium when compared to dietary guidelines for Americans [17]. These nutritional deficits and excesses can exacerbate SCI-related health complications.

Nutritional interventions are crucial in combating the myriad of secondary health conditions associated with SCI, including neurogenic obesity, type 2 diabetes, dyslipidaemia, and other obesity-related comorbidities [18]. Promising dietary strategies encompass high-protein diets, intermittent fasting, balanced nutrition combined with physical conditioning and electrical stimulation, and targeted supplementation (e.g., alpha-lipoic acid, creatine, vitamin D, cranberry derivatives, and probiotics) [19]. Moreover, maintaining a healthy weight through proper nutrition significantly reduces the risk of diabetes, metabolic syndrome, cardiovascular disease, and skin breakdown in this vulnerable population [20].

Despite the clear importance of nutrition in SCI management, developing comprehensive, evidence-based nutritional guidelines remains challenging. This is due to the relative novelty of nutrition focus in SCI research, the unique physiological changes post-injury, and the methodological challenges inherent in conducting robust nutritional studies in this population [21,22]. Often, experts must extrapolate from general population data, which may not accurately reflect the specific needs of individuals with SCI. Furthermore, SCI nutrition studies frequently suffer from insufficient statistical power due to recruitment difficulties, high attrition rates, and limited funding for large-scale, multi-centre trials [23].

To the authors’ knowledge, no result of a systematic, international priority setting for nutrition research has been published so far. To overcome these obstacles and advance the field, a strategic, prioritized approach to SCI nutrition research is imperative. By systematically identifying and consensus-building around key research priorities, we can focus limited resources on areas with the greatest potential impact on health outcomes and quality of life for individuals with SCI.

This study aims to identify and achieve consensus on priorities for nutrition research in adults with SCI, providing a crucial roadmap to guide international research efforts. By aligning research focus with stakeholder-identified priorities, we can accelerate progress in this critical yet under-researched area, ultimately improving the lives of individuals living with SCI worldwide.

## Methodology

### The Delphi processes

**Fig 1:**
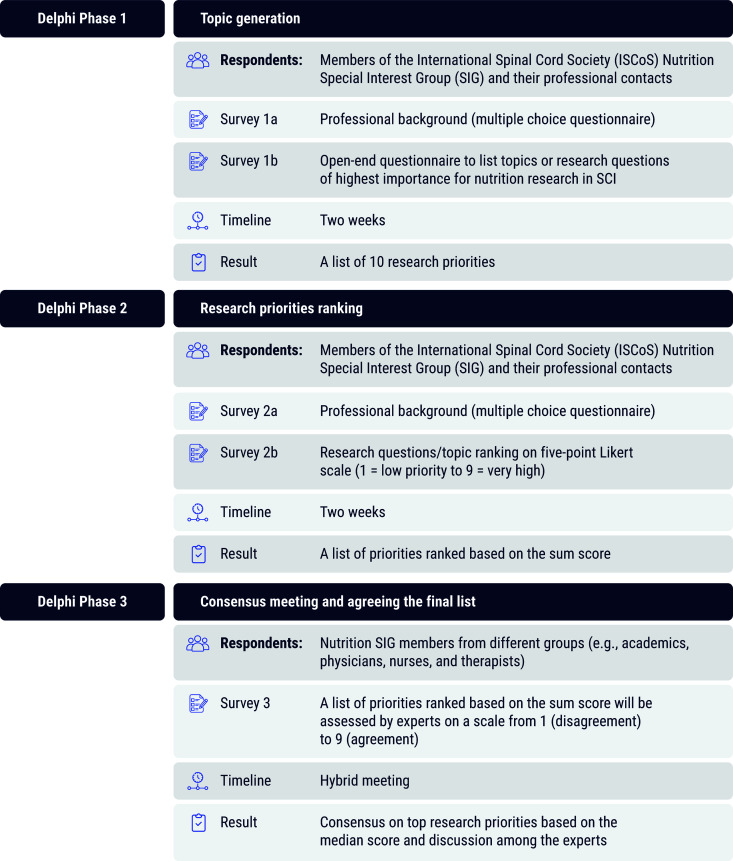
The study flowchart. The Delphi process will be conducted in three phases. Phase 1 focuses on topic generation through an open-ended survey to members of the ISCoS Nutrition Special Interest Group (SIG) and their professional networks, resulting in a list of 10 preliminary research priorities. Phase 2 involves ranking these priorities using a Likert scale to determine their relative importance, leading to a list ranked by sum scores. Phase 3 consists of a hybrid consensus meeting with Nutrition SIG members to finalize the top research priorities based on median scores and expert discussion.

The study conduct and report will follow the Conducting and Reporting of DElphi Studies (CREDES) recommendations [24]. To identify research priorities in the field of nutrition among SCI population, we will perform a modified electronic Delphi (e-Delphi) process to facilitate wider (international) participation. The study has not yet started and will be conducted between April and October 2025. It will comprise a systematic, two-round, web-based, eDelphi questionnaire among diverse stakeholders involved either in clinical care or SCI research, followed by hybrid consensus meeting (Fig 1). Results are expected by the end of 2025. The project has been granted the ethical approval from the School of Health & Psychological Sciences at City St George’s, University of London Ethics board (ETH2425−0192).

### Participants/Respondents

Health professionals who are members of the International Spinal Cord Society (ISCoS) Nutrition Special Interest Group (SIG) and their professional contacts are eligible to participate in two rounds of anonymous web-based surveys. Participation in Phase 3 (consensus meeting) is reserved for Nutrition SIG members only. To ensure that participants have sufficient expertise to contribute meaningfully to the Delphi process, while balancing inclusivity with subject matter rigor, eligibility requires meeting at least one of the following criteria:

•Have at least two years of experience in SCI research
•Have at least two papers published in the field of clinical nutrition.
•Hold a senior academic position (i.e., professorship, group leader)
•Have at least five years of experience in clinical or academic work

Eligible members of the ISCoS Nutrition SIG will be invited to participate via direct contact, newsletters and through social media. Snowball sampling of close contacts (meeting the above-mentioned criteria) will be applied to increase the sample size. Participants will provide informed consent after reading an approved Participant Information Sheet and having the opportunity to ask questions and receive satisfactory response (in person, online or electronically). Participants will complete the study consent form online via a secure, web-based platform.

### Delphi phase 1: The topic generation

The Nutrition SIG members and eligible professional contacts will be asked to fill-out the online survey using Qualtrics online survey software. The participants will be asked to provide information on their professional background and to generate a list of all possible topics or research questions which they consider important for nutrition research among SCI population. Participants will be asked to consider all aspects of nutrition research such as research methodology, standardized data collection, target population, scientific methodology, health outcomes etc. Three members of the research team (MG, SH and HBS) will review the generated items, merge similar topics and draft a list of unique research priorities. This list will be discussed in an online SIG meeting and agreed among the experts and the final list of 10 research topics will be developed into the online survey 2.

### Delphi phase 2: Online survey on ranking the top 10 research priorities

Respondents will provide information on their professional background and will be asked whether they have participated in Phase 1 of the study. The participants will be asked to rank top 10 research priorities on a Likert scale of 1–9 (where 9 indicates high priority and 1 indicates low priority), based on their own experiences and based on the relevance, feasibility and impact of research outcome. They will also be asked to provide a rationale for their top three research priorities. The survey will be live for two weeks. After the closure of the survey, the questions will be ranked based on the sum score. The rankings will be reviewed for the whole sample and then separated by stakeholder groups to identify any striking differences between stakeholder groups that may have skewed the whole sample ranking.

### Delphi phase 3: Consensus meeting and agreeing the final list

A consensus meeting will be held in hybrid form. Nutrition SIG members from different groups (e.g., academics, physicians, nurses, and therapists) who participated in the first two phases will be invited to attend. An experienced facilitator will moderate the meeting following an agreed agenda. The hybrid consensus meeting will be recorded. After the meeting, these will be manually transcribed into Microsoft Word document by “an Admin Person”, with pseudonyms for anonymity, that is, HP1, HP2, PT1, PT2. Participants names will be excluded from reports, to ensure the information cannot be tracked back to the participants. Once the transcription and analysis are completed, the recording will be destroyed. Attendees will not be required to provide additional consent to participate in the meeting. The proposed list of questions will be discussed individually, and attendees will use the Mentimeter® or PollEV to anonymously vote for items to be included in the final list. Each research priority will be rated by all experts on a scale from 1 to 9 (1 = disagreement, 9 = agreement). A median score will be calculated, and results will be classified as disagreement (median score ranging from 1 to 3), indecision (median score ranging from 4 to 6) and agreement (median score ranging from 7 to 9). To formally define consensus, an additional criterion will be applied: an item will be considered to have reached consensus agreement if it receives a median score of 7–9 and at least 70% of participants rate the item within that same range. Similarly, an item will be considered to have consensus disagreement if it has a median score of 1–3 and at least 70% of responses fall within that range. Items that do not meet these thresholds will be classified as uncertain or lacking consensus and may be reconsidered for voting. Discussions on the final wording of the top list of questions will be continued by email and would be discussed in virtual meetings as necessary, and the final list will be agreed upon within four weeks.

### Strategy for data synthesis

Descriptive statistics will be used to describe the survey respondents and consensus meeting’s participants. Both surveys will be developed using Qualtrics, and all responses collected over encrypted SSL (TLS) connections. All responses will be transferred to City St George’s, University of London approved secure servers (i.e., MS OneDrive for Business) and analysed using STATA/ SPSS. Mentimeter®/ PollEV votes in consensus meeting will be collected anonymously, and the results downloaded and saved on the same OneDrive servers. Qualtrics and PollEV are, secure software meeting the Information Assurance threshold tests for research projects and approved by City St George’s, University of London, Information Systems leads.

## Expected results and dissemination

This Delphi study is anticipated to yield several significant outcomes that will advance the field of nutrition research in SCI. First, we expect to generate a comprehensive, ranked list of 10–15 key research priorities, reflecting the consensus of international experts with established experience in clinical nutrition and SCI. This prioritized research agenda will likely reveal areas of agreement and potential divergence among participating professionals with interdisciplinary background, offering insights into shared priorities and areas requiring further dialogue. While the focus on members of the ISCoS Nutrition Special Interest Group (SIG) and their professional networks ensures a high level of subject-matter expertise, we acknowledge that this approach may limit broader representativeness. In particular, excluding patients, caregivers, and other stakeholder groups means that the identified priorities will primarily reflect the perspectives of health professionals. This decision was intentional, as clinical and research expertise in SCI nutrition requires a deep understanding of complex physiological, metabolic, and clinical contexts. Nonetheless, we recognize the value of including lived experiences and lay perspectives in shaping a more holistic research agenda, and future work may expand to incorporate patient and caregiver input through complementary methods such as focus groups or patient advisory panels.

Additionally, the study is expected to highlight critical knowledge gaps in SCI nutrition, pinpointing where evidence is lacking, and research is urgently needed. The findings will serve as a valuable tool for funding bodies and research institutions, guiding resource allocation towards the most impactful areas. We anticipate identifying opportunities for international collaboration, fostering partnerships to tackle complex, high-priority research questions. Moreover, the results are likely to inform clinical practice guidelines by emphasizing areas where nutritional interventions could significantly improve patient outcomes. Insights from the study may also lead to policy recommendations for SCI care and rehabilitation, underscoring the essential role of nutrition in comprehensive management. Overall, we expect this study to provide clear direction for future research efforts, potentially catalysing new studies and clinical trials in high-priority areas while raising awareness about the importance of nutrition in SCI care among both the scientific community and the public.
